# More are better, but the details matter: combinations of multiple Fresnel zone plates for improved resolution and efficiency in X-ray microscopy

**DOI:** 10.1107/S1600577518007208

**Published:** 2018-06-17

**Authors:** Kenan Li, Chris Jacobsen

**Affiliations:** a Applied Physics, Northwestern University, Evanston, IL 60208, USA; b Advanced Photon Source, Argonne National Laboratory, Argonne, IL 60439, USA; c Department of Physics and Astronomy, Argonne National Laboratory, Argonne, IL 60439, USA; d Chemistry of Life Processes Institute, Northwestern University, Evanston, IL 60208, USA

**Keywords:** Fresnel zone plates, X-ray microscopy, X-ray optics

## Abstract

Fresnel zone plates used for X-ray nanofocusing face high-aspect-ratio nanofabrication challenges in combining narrow transverse features (for high spatial resolution) along with extended optical modulation along the X-ray beam direction (to improve efficiency). The stacking of multiple Fresnel zone plates along the beam direction has already been shown to offer improved characteristics of resolution and efficiency when compared with thin single zone plates. Using multislice wave propagation simulation methods, here a number of new schemes for the stacking of multiple Fresnel zone plates are considered. These include consideration of optimal thickness and spacing in the axial direction, and methods to capture a fraction of the light otherwise diffracted into unwanted orders, and instead bring it into the desired first-order focus. The alignment tolerances for stacking multiple Fresnel zone plates are also considered.

## Abstract 2

The properties of stacking multiple Fresnel zone plates together at intermediate distances are considered. One can enhance the nanofocusing efficiency, and other characteristics, by careful choice of design parameters.

## 1. Introduction   

**Figure 1:**
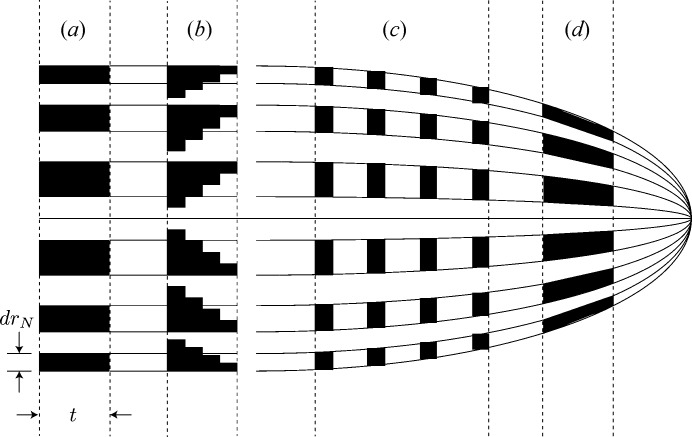
Zone plates modulate the phase and magnitude of a transmitted wave so as to direct it to a focus. This can be done in several ways, using magnitude and/or phase modulations on alternating zones of half-wavelength optical path difference to the focus. A simple Fresnel zone plate (*a*) has an outermost zone width of 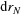 and a thickness *t*; it applies a constant magnitude reduction or phase shift across each zone, while a blazed zone plate (*b*) puts a staircase approximation of a phase ramp across the zone. One can stack several thin Fresnel zone plates (*c*) to manipulate the wave at several locations leading to the focus, or use thicker zones (*d*) which are individually tilted to meet the Bragg grating condition or even curved in a volume hologram approach to produce the converging wavefield.

Fresnel zone plates are widely used as the nanofocusing optic in X-ray microscopes (Sakdinawat & Attwood, 2010 ▸; Attwood & Sakdinawat, 2017 ▸). They focus beams or image specimens with a Rayleigh resolution of 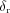 = 1.22d*r*_*N*_/*m* where 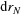 is the width of the finest outermost zone and *m* is the diffractive order used for focusing. Their first-order focusing efficiency depends on the refractive index 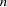 = 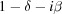 of the zone material (Henke *et al.*, 1993 ▸), with a scalar diffraction efficiency 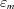 for a zone plate thickness *t* given by (Kirz, 1974 ▸) 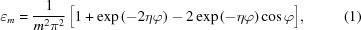
where 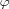 = 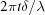 and 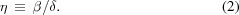
The efficiency reaches a maximum at a thickness 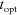 satisfying the condition 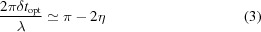
or 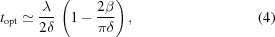
which for gold gives 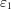 = 8.7% at 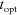 = 98 nm when using 0.5 keV soft X-rays, and 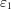 = 32.7% at 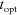 = 2.0 µm when using 10 keV hard X-rays. Thus one can see that efficient high-resolution multi-keV X-ray focusing requires the fabrication of high-aspect-ratio structures with narrow zone width 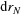 but large thickness *t* [see Fig. 1(*a*) ▸]. There is in fact much progress in fabricating conventional Fresnel zone plates with high aspect ratios (Schneider *et al.*, 1995 ▸; Spector *et al.*, 1997 ▸; Jefimovs *et al.*, 2007 ▸; Werner *et al.*, 2014 ▸; Chang & Sakdinawat, 2014 ▸; Mohacsi *et al.*, 2017 ▸; Li *et al.*, 2017*b* ▸). However, even if technological limits on high-aspect-ratio nanofabrication were removed, there remain fundamental limits: as zones increase in thickness, they begin to need to be tilted to match the Bragg condition (Maser & Schmahl, 1992 ▸) or even curved to match the converging wave as in a volume hologram (Pennington & Lin, 1965 ▸; Kogelnik, 1969 ▸; Goodman, 2005 ▸), as shown in Fig. 1(*d*) ▸.

An alternative approach to a monolithic three-dimensional optical structure is to stack multiple separate zone plates together. When doing so, one must pay attention to how the separation distances 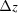 compare with the depth of focus. The depth of focus 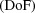 of a Fresnel zone plate can be expressed as twice the depth resolution 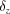 or (Wang *et al.*, 2000 ▸) 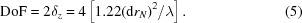
When multiple zone plates are located well within a depth of focus along the wavefield propagation direction, one can treat the net effect as being due to a zone plate with the combined thickness *t* as has been demonstrated (Shastri *et al.*, 2001 ▸; Maser *et al.*, 2002 ▸; Snigireva *et al.*, 2007 ▸; Kagoshima *et al.*, 2011 ▸; Feng *et al.*, 2007 ▸; Aristov *et al.*, 2007 ▸; Mohacsi *et al.*, 2014 ▸, 2017 ▸; Rehbein *et al.*, 2015 ▸). However, this close proximity is often challenging to achieve in practice, leading to an alternative approach of stacking multiple zone plates with separations larger than 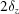 provided each individual zone plate is designed to focus to a common location (Vila-Comamala *et al.*, 2013 ▸; Gleber *et al.*, 2014 ▸). This has been studied within the approximation that each zone plate is optically thin, so that one can apply each zone plate’s optical modulation to a wavefield, and then propagate it by a distance of 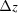 to the plane of the next zone plate to model the net focusing effect.

In this paper, we look in further detail at the optical properties of multiple stacked Fresnel zone plates. We make use (when necessary) of the multislice propagation technique (Cowley & Moodie, 1957 ▸) which can replicate (Li *et al.*, 2017*a* ▸) the results of coupled-wave theory which is otherwise required (Maser & Schmahl, 1992 ▸; Schneider, 1997 ▸; Schneider *et al.*, 2008 ▸) when studying very high aspect ratio zone plates. We consider for the first time the following features of stacked zone plates:

(i) While previous calculations (Vila-Comamala *et al.*, 2013 ▸) and experiments (Gleber *et al.*, 2014 ▸) considered intermediate distance stacking with zone plates with fixed outermost zone width 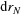 and adjustable diameter *d*, we consider the three cases of fixed 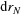, fixed *d* and fixed zone number *N* in §2.

(ii) We examine in detail the exit wave from the last of a stacked set of Fresnel zone plates, and show in §3 that with multiple zone plates one begins to produce an effect like that of blazed zone plates but without the tradeoff of minimum linewidth otherwise required.

(iii) We show in §4 that the Talbot effect can be used to understand that there are optimum separation distances between stacked zone plates.

(iv) In §5 we consider ‘complementary’ zone plates where the positions of ‘open’ and ‘filled’ zones are reversed in some elements of a stacked combination, and show that this can reduce the effect of sidelobes off of the focus which can be advantageous for applications such as trace-element mapping using X-ray fluorescence.

(v) We consider ‘refocusing’ zone plates in §6, where some of the negative or diverging focal-order light from an upstream zone plate is captured and brought back into the positive first-order focus.

In addition to the above, in the supporting information we consider alignment tolerances of stacked zone plates as already discussed in the references cited, but provide more detail on misalignment effects.

## 2. Selecting multiple zone plate parameters   

For larger separation distances between individual zone plates, the relationship between diameter *d*, outermost zone width 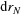 and zone number *N* of 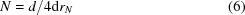
and the focal length of 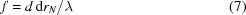
indicate that one has a choice in fixing any one of three parameters in order to adjust the *i*th zone plate to have a focal length 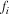 so as to focus at the common location. These strategies are as follows:

(i) Fixed outermost zone width 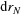: in this case, the diameter is adjusted according to 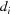 = 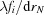 and the number of zones is adjusted to 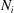 = 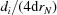.

(ii) Fixed diameter *d*: in this case, the outermost zone width is adjusted according to 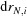 = 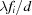 and the number of zones is adjusted to 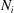 = 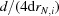.

(iii) Fixed zone number *N*: in this case, the diameter is adjusted according to 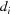 = 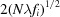 and the outermost zone width is adjusted to 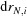 = 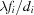.

While the fixed 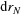 approach has been studied previously (Vila-Comamala *et al.*, 2013 ▸; Gleber *et al.*, 2014 ▸), the fixed *d* and fixed *N* approaches have not.

Because many nanofabrication processes have limits on the achievable aspect ratio 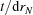, when considering approaches that vary the outermost zone width 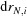 we have also chosen to adjust the thickness of the *i*th zone plate according to 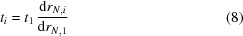
for the fixed diameter *d* and fixed zone number *N* choices. In order to make clear the differences between these strategies, we show in Table 1 ▸ the parameters that result when four zone plates are used at 10 keV with a very large separation of 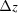 = 1 mm between each zone plate.

**Figure 2:**
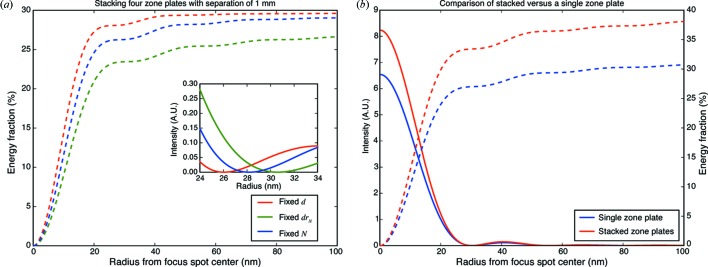
The stacking of multiple thinner zone plates can lead to higher optical performance than can be obtained with a thicker single zone plate. On the left (*a*) is show the focusing efficiency (in terms of integrated energy fraction as a function of radius from the focal spot center) and, as an inset, the focused intensity profile for the three zone-plate design schemes and parameters outlined in Table 1 ▸. This is for the case of zone plates separated by a very large distance of 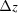 = 1000 µm relative to the focal length of 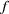 = 9074 µm for the first, upstream zone plate. The inset shows that the strategy of fixed diameter *d* gives a higher Rayleigh resolution (smaller radius for the first minimum of the intensity distribution) than the strategies of fixed outermost zone width 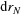 or fixed zone number *N*. On the right (*b*) is shown the integrated energy fraction for a single 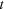 = 2000 nm-thick zone plate *versus* four 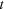 = 500 nm-thick zone plates separated by 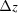 = 50 µm, and designed according to the fixed diameter *d* strategy. If multiple thinner zone plates can be aligned with sufficient accuracy, they can offer higher overall focusing efficiency (though in this case the separation between the zone plates is so small that they all have essentially the same numerical aperture, so there is no spatial resolution gain).

To understand the consequences of these different strategies, in Fig. 2 ▸ we show the efficiency and spatial resolution values that result. The case of fixed diameter *d* yields the highest efficiency and resolution, even though the downstream zone plates are assumed to have lower thickness 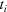 and thus lower individual diffraction efficiency. In the case of fixed diameter *d*, the downstream zone plates capture a larger fraction of the zero-order undiffracted light passing through upstream zone plates, and the final zone plate has a smaller outermost zone width 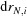 which contributes to a higher spatial resolution. This increase in area more than compensates for the decrease in thickness 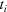. In contrast to this case, the fixed outermost zone width 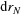 case has the smallest diameter 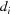 for the later zone plates. A factor not considered in the efficiency or energy fraction shown in Fig. 2 ▸ is the spectral bandwidth, which should be limited (Thieme, 1988 ▸) to 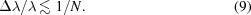
In the case of fixed *d*, the change from 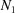 = 450 to 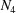 = 672 zones would reduce the acceptable spectral bandwidth by 33.0% relative to the fixed zone number *N* case; in the fixed outermost zone width 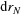 case one would not gain from the smaller value of 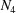 = 301 because the spectral bandwidth would still be limited by 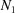 = 450. The choice of fixed diameter *d* or fixed zone number *N* therefore depends on the degree to which spectral bandwidth acceptance can be controlled, since in many cases zone plate microscopes are operated with crystal monochromators with values of 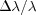 far smaller than what equation (9) would require.

## 3. Multiple zone plate exit waves   

**Figure 3:**
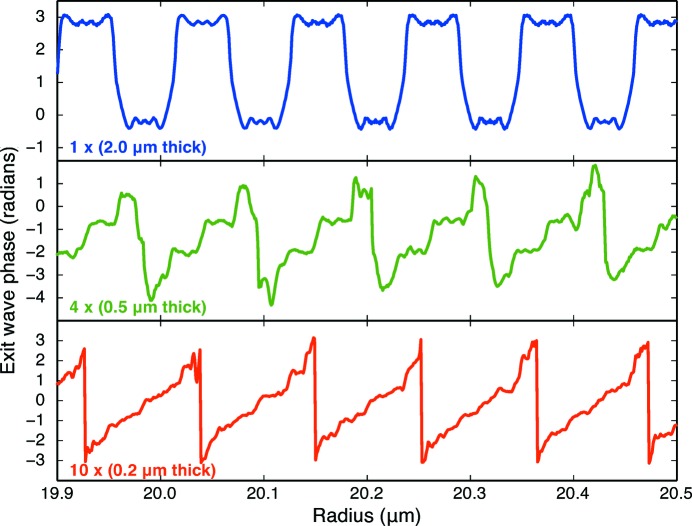
Phase profile of the exit wave from (top) a single 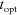 = 2.0 µm-thick zone plate of gold for 10 keV X-ray focusing, (middle) four 0.5 µm-thick zone plates, and (bottom) ten 0.2 µm-thick zone plates. In each case the phase of the exit wave from the final downstream zone plate is shown. By using multiple thin zone plates, one can better approximate the phase profile of a blazed zone plate (Fig. 1*b* ▸).

In near-field zone plate stacking, the optical effect of each zone plate is simply superimposed on the incident wavefield with no interceding propagation-based wavefield evolution. In non-near-field stacking, simulations (Vila-Comamala *et al.*, 2013 ▸) and experiments (Gleber *et al.*, 2014 ▸) have calculated the intensity profile near the focal region. In Fig. 3 ▸, we show for the first time the nature of the exit wave from the last of a set of stacked zone plates. (This exit wave then converges to produce the focal spot.) When multiple stacked zone plates are used, the exit wave from multiple stacked zone plates evolves towards that of a blazed zone plate (Fig. 1*b* ▸). Because of this, one can achieve a higher diffraction efficiency with a set of separated stacked zone plates than would be expected simply from the sum of the zone plate thicknesses and equation (1).

A staircase approximation to single-optic blazed zone plates has been realized by using multiple overlaid lithography steps to produce a single optical structure with improved focusing efficiency (Krasnoperova *et al.*, 1993 ▸; Di Fabrizio *et al.*, 1994 ▸, 1999 ▸; Yun *et al.*, 1999 ▸). However, if *k* lithographic overlays are used to produce a zone profile with *k* stair steps, the finest transverse feature size in the last stair step must be 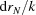 to achieve a net width 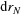 of the finest outermost half of the blazed structure. In other words, if the finest transverse feature size that a given lithographic process can produce is *a*, then the finest zone half period is *ka* rather than having 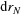 = 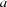. In this case the increase in efficiency of using *k* steps to produce a staircase approximation of a blazed zone plate comes at a cost in achievable spatial resolution.

Separated stacked zone plates can avoid this undesirable tradeoff. In Fig. 3 ▸, we show the phase of the wavefield exiting the final downstream zone plate for the case of a single 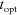 = 2000 nm-thick [equation (4)] zone plate of gold for use at 10 keV for which equation (1) gives a diffraction efficiency of 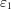 = 32.7%, as well as the exit waves for four zone plates each with 500 nm thickness, and ten zone plates each with 200 nm thickness (in all cases the separation distance was 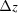 = 10 µm). The wavefield propagation through each individual zone plate was calculated using a multislice approach (Cowley & Moodie, 1957 ▸; Li *et al.*, 2017*a* ▸). As was noted, the ten zone plate case produces a last-zone-plate exit wave strongly resembling what one would have from a blazed zone plate.

**Figure 4:**
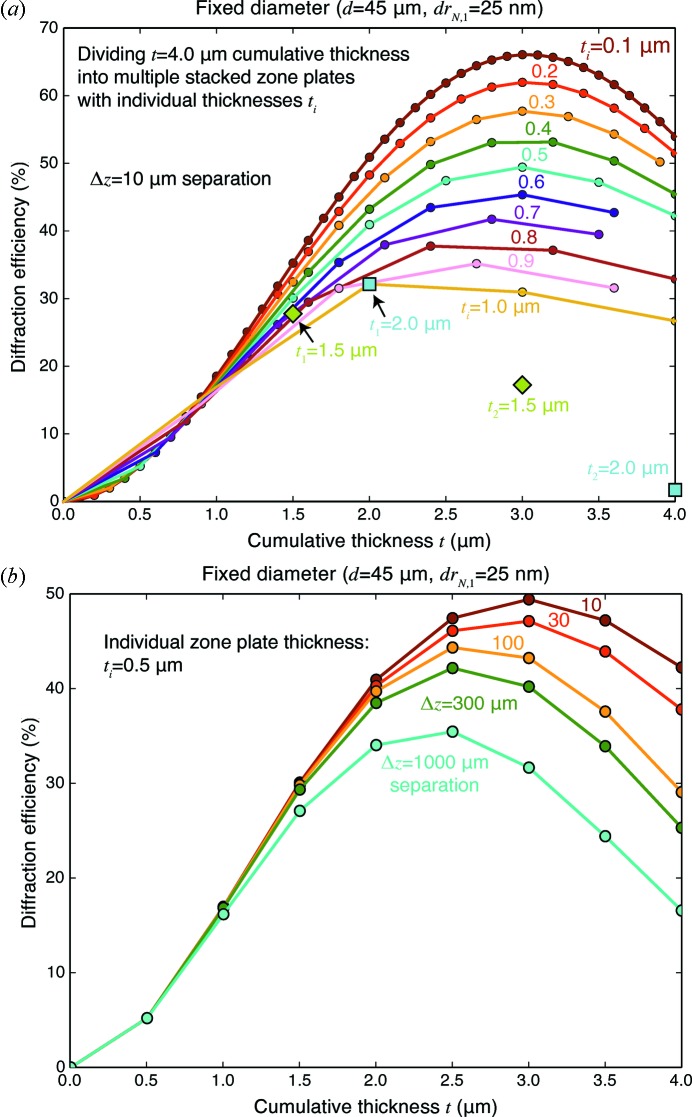
While there might be practical limitations to the number of zone plates 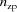 that can be stacked together, one can obtain gains in first-order diffraction efficiency 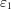 that go well beyond the simple thin zone plate expression of equation (1). In (*a*) we show the efficiency as a function of individual zone plate thickness 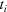 and cumulative thickness *t*, where 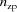 = 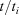 zone plates are used with with a separation of 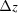 = 10 µm (for 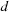 = 45 µm, and 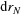 = 25 nm for the first zone plate at 10 keV). A single gold zone plate with the optimum thickness 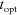 = 2.0 µm would give 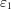 = 32.7%, whereas much higher efficiencies can be obtained by using many more zone plates with slightly higher cumulative thickness. In (*b*) we show how the stacking of 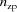 = 8 zone plates, each with a thickness 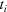 = 0.5 µm, leads to differences in diffraction efficiency as one changes the separation distance 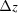 between zone plates. Smaller separation distances 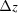 are preferable but might be impractical, but even with larger separation distances like 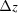 = 1000 µm one can still obtain an efficiency of 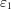 = 36% if 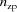 = 5 zone plates are used. All calculations were for gold zone plates at 10 keV.

In order to explore this further, we first consider the case of the number of zone plates to be used and the resulting diffraction efficiency. While the optimum thickness for a single zone plate of gold at 10 keV is 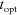 = 2.0 µm, in Fig. 4 ▸ we consider cumulative zone plate thicknesses as high as 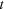 = 4.0 µm while in fact using multiple zone plates with individual thicknesses 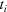 ranging from 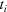 = 0.1 µm (so that 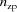 = 40 such zone plates would have an accumulated thickness of 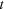 = 4.0 µm if they had the same design parameters) to 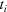 = 2.0 µm (so that only 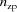 = 2 zone plates would be stacked). In all cases, a separation distance of 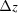 = 10 µm was used. As this figure shows, one can obtain a diffraction efficiency of 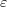 = 66% if one uses 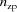 = 30 zone plates that are each only 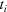 = 0.1 µm thick at 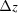 = 10 µm spacing, where each is designed according to the fixed diameter *d* strategy described in §2. Even if fewer zone plates are used with a cumulative thickness limited to 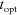 = 2.0 µm, one still sees gains over single zone plates: for example, 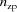 = 4 zone plates each with 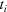 = 0.5 µm give an efficiency of 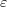 = 41.5% while if one uses 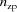 = 10 zone plates with 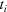 = 0.2 µm the efficiency is 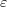 = 48.9%.

Fig. 4(*a*) ▸ used a constant separation distance of 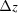 = 10 µm between zone plates, which is in practice a very small separation distance. Fig. 4(*b*) ▸ shows how the performance of multiple zone plates (each with 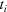 = 500 nm thickness) changes as one increases the separation distance 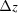. As can be seen, smaller separation distances are preferred, but even with 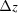 = 100 µm one can obtain a first-order diffraction efficiency of 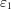 = 44.8% by using 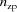 = 5 zone plates.

## 4. The Talbot effect and zone plate spacing   

**Figure 5:**
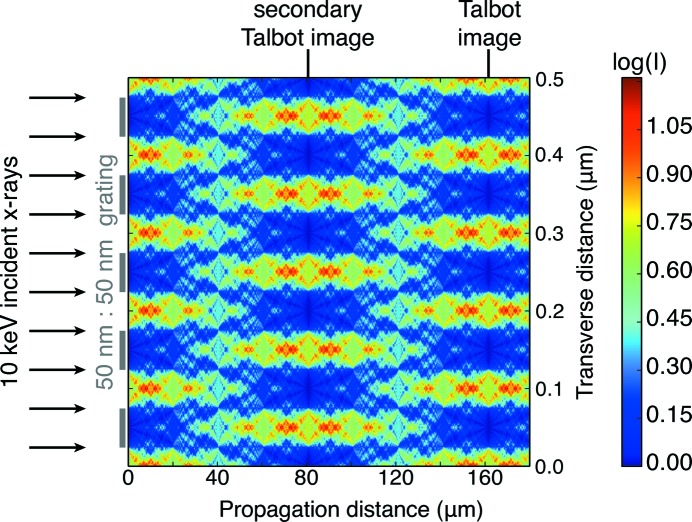
Illustration of the Talbot effect. When a periodic diffraction grating is illuminated by a plane wave, the image of the grating is repeated at a Talbot distance of 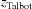 = 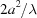 [equation (10)] where *a* is the period of the diffraction grating. For a grating period of 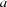 = 100 nm, and 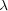 = 0.124 nm (corresponding to 10 keV X-rays), the Talbot distance is 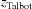 = 161 µm. The grating here was assumed to be fully absorptive.

The Talbot effect (Talbot, 1836 ▸; Lord, 1881 ▸) involves the replication of a transmission grating pattern of period *a* at a Talbot distance
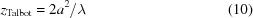
when zero and first diffraction orders constructively interfere (Fig. 5 ▸). This suggests that there might be an optimum separation distance 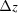 for stacked zone plate. Because the local grating period in a Fresnel zone plate varies constantly with radius, it is less clear whether the Talbot effect should apply based on the period 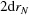 of the finest zone width, or on the period 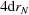 of the zones at half the radius, or whether it is ‘washed out’ by the variation in zone width.

**Figure 6:**
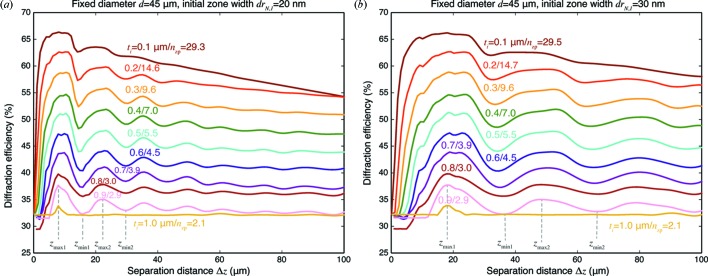
Diffraction efficiency 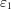 *versus* separation distance 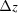 for different thicknesses 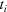 of individual zone plates, and for two different values of outermost zone width: 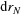 = 20 nm (*a*) and 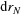 = 30 nm (*b*). For each individual zone thickness 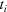 and separation distance 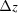, the number of stacked zone plates 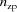 was chosen to give maximum efficiency, as shown in Fig. 4(*b*) ▸, and the average value of 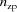 corresponding to a particular value of 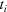 is indicated. As can be seen, there is a pattern of maxima and minima in the efficiences as a function of separation distance 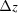, with the first two maxima denoted by 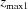 and 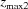, and the first two minima denoted by 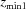 and 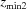. These maxima and minima are compared with the Talbot distance [equation (10)] for the outermost zones in Fig. 7 ▸.

While Fig. 4(*b*) ▸ showed the effect of only a few different separation distances 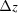, in Fig. 6 ▸ we show the effect of a much larger number of more finely varied separation distances. In this figure, for each value of individual zone plate thickness 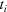 and separation distance 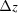, the optimum number 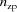 of individual zone plates was chosen as is shown in Fig. 4(*a*) ▸ and the average value of 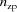 is shown for each thickness 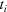. Clearly, we see maxima and minima of efficiencies with respect to stacking separations, and a dependence on outermost zone width 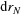.

**Figure 7:**
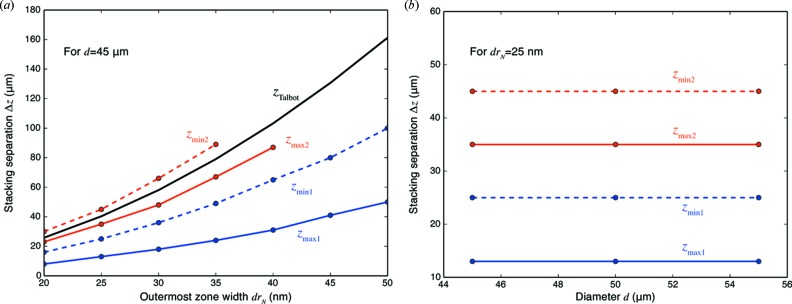
Positions of the efficiency maxima 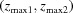 and minima 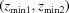 shown in Fig. 6 ▸ as a function of varying outermost zone width 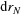 (*a*) or diameter *d* (*b*). This was done for an individual zone plate thickness of 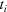 = 0.9 µm. Clearly the separation distances 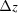 show maxima and minima that scale with outermost zone width 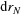 rather than diameter *d*. Also shown on the left is the Talbot distance 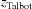 based on equation (10) for a grating with a period 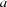 = 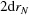 determined by the outermost zone width. The efficiency maxima and minima scale with 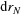, but the ideal separation distance 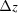 corresponding to 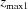 is at about one-quarter of the distance 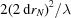.

In order to better compare the separation distances leading to the first two maxima (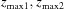) and minima (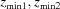) in Fig. 6 ▸, in Fig. 7 ▸ we show these extrema both as a function of varying the outermost zone width 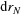 in Fig. 7(*a*) ▸, and the zone plate diameter *d* in Fig. 7(*b*) ▸. Obviously the efficiency maxima and minima depend on outermost zone width 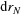 rather than diameter *d*. In Fig. 7(*a*) ▸, we also indicate the Talbot distance 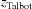 of a grating with a period 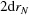 corresponding to the outermost zone width. While the positions of the maxima and minima show the expected scaling with 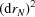, the separation distance 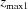 corresponding to the first efficiency maximum is about one quarter of the value 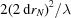 that one would predict from equation (10) based on the outermost zone width. As shown in Fig. 5 ▸, at this distance a constant-period grating shows an interference pattern at half the period of the grating, and with a contrast inversion.

## 5. High diffraction orders and complementary zone plates   

**Figure 8:**
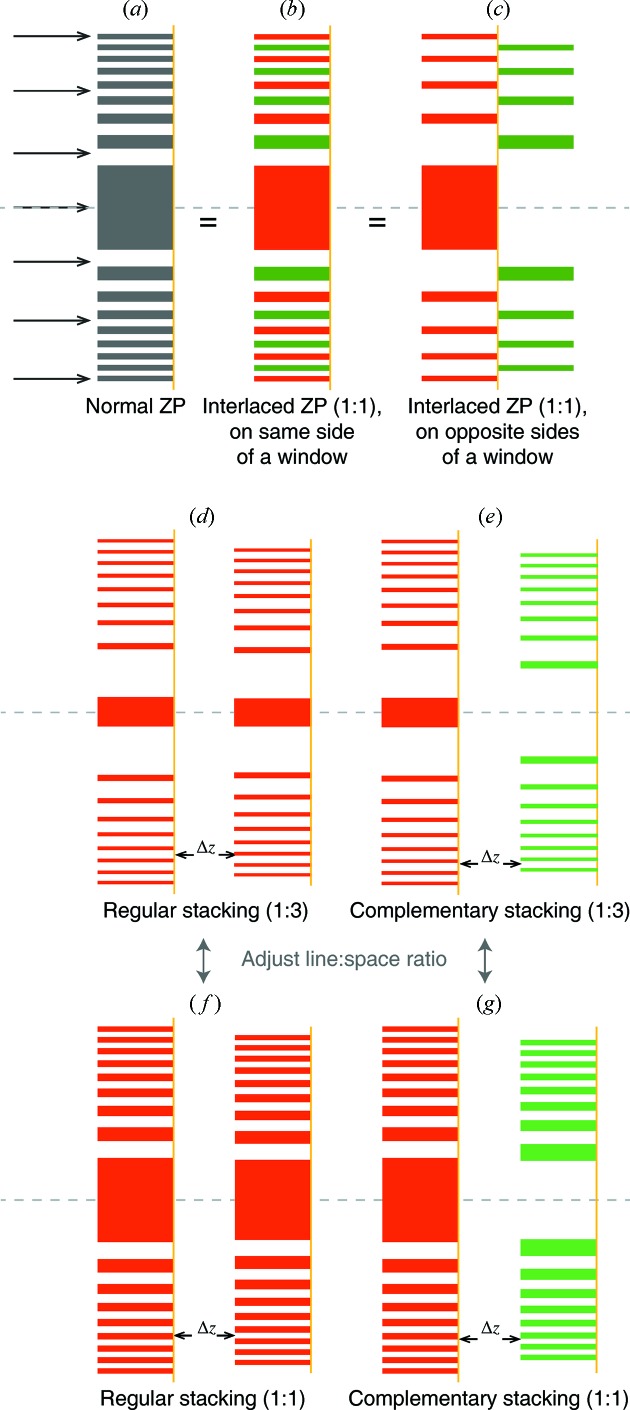
A normal Fresnel zone plate with 1:1 line:space ratio (*a*) can be constructed by interlacing two fabrication processes, either on the same side of a thin window (Chao *et al.*, 2005 ▸) (*b*) or on opposite sides (Mohacsi *et al.*, 2017 ▸) (*c*). Both of those interlaced processes reduce the limitations produced by the proximity effect in electron beam lithography in the separate fabrication processes. The proximity effect is reduced further if one fabricates zones with a line:space ratio of 1:3 and uses them in the second diffraction order. One can also use complementary zone plates with an opposite pattern of material-filled/open zones. That leads to several options in zone plate stacking: stacking with 1:3 line:space ratio with a regular (*d*) or complementary (*e*) second zone plate, or with 1:1 line:space ratio with a regular (*f*) or complementary second zone plate. These various combinations give different properties for focusing efficiency and focal spot sidelobes, as shown in Fig. 9 ▸.

In nanofabrication using electron beam lithography, transverse spreading of the electron beam in the photoresist (the proximity effect) can complicate the fabrication of dense narrow-linewidth structures such as the zones in a Fresnel zone plate. For this reason, a variety of interlacing approaches have been used in which every other zone is written in one operation, with a subsequent identical operation used to write the alternating zones either on the same side of a thin window (Chao *et al.*, 2005 ▸) as shown in Fig. 8(*b*) ▸, or on the opposite side (Mohacsi *et al.*, 2017 ▸) as shown in Fig. 8(*c*) ▸. Another way to reduce the limitations of the proximity effect is to write narrower zones in a low-density template, and use atomic layer deposition (ALD) to deposit high-density material on these templates in a process known as zone doubling (Jefimovs *et al.*, 2007 ▸). Yet another approach is to consider the fabrication of zone plates with line:space ratios other than 1:1 where the separation distance between written structures can be increased (again reducing the proximity effect in electron beam lithography), while working in higher diffraction orders (Schneider, 1997 ▸).

Inspired by these approaches, in Fig. 8 ▸ we consider several options for zone plate stacking:

(i) The first of these is the use of line:space ratios with values such as 1:3, giving more space between lithographically patterned zones and thereby reducing the limitations set by the proximity effect. With a 1:1 zone plate, the outermost zone period is 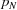 = 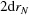 and the Rayleigh resolution is 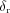 = 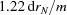. With other values of the line:space ratio, it is better to base the Rayleigh resolution expression using the outermost zone period 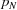, giving 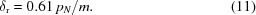
When the line:space ratio in a grating is changed from 1:1 to other values, the energy distribution into various diffractive orders is modified. For example, a 1:3 zone plate operated in the 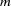 = 2 diffraction order can in principle have twice the spatial resolution for a given period 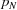 as shown in equation (11), and there can be specific thicknesses and line:space ratios which deliver high focusing efficiency into the 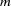 = 2 order as calculated using coupled wave theory (Schneider, 1997 ▸). By working in the second diffraction order, one obtains a factor of two improvement in spatial resolution compared with the minimum zone width.

(ii) The second of these is the use of complementary zone plates. In the normal case for a Fresnel zone plate, the zeroth or central zone is filled with material up to a radius 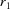 as given by 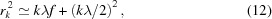
after which one alternates between open and material-filled zones such that the next material-filled zone is bounded by 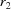 and 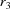. However, in a complementary zone plate the central zone is open and the first material-filled zone is bounded by 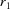 and 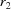. Complementary zone plates do not by themselves provide any reduction in the proximity effect, but they can be used with any line:space ratio including 1:1 or 1:3.

Variation of the line:space ratio and the use of regular or complementary zone plates give us additional options in stacked zone plate design. In addition, when using separated stacked zone plates rather than the interlacing schemes of Figs. 8(*b*) or 8(*c*) ▸, one gains the ability to use processes that are difficult to interlace on one window. One of these is metal-assisted chemical etching (MACE) to produce extremely high aspect ratio zone-doubling template structures for ALD (Chang & Sakdinawat, 2014 ▸; Li *et al.*, 2017*b* ▸).

**Figure 9:**
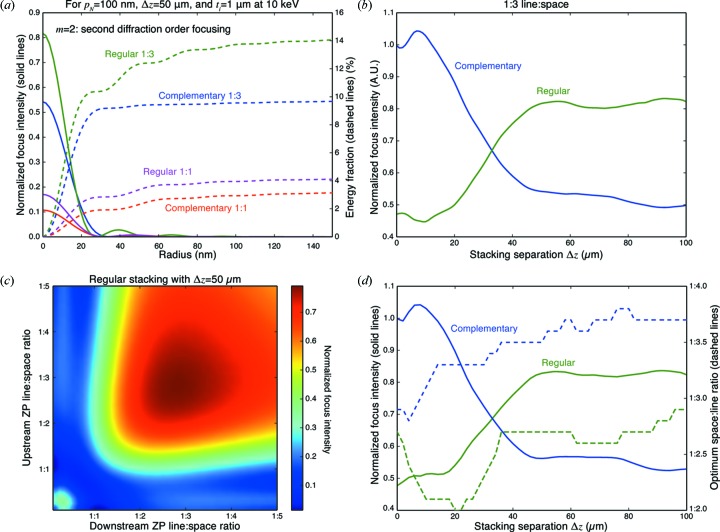
Stacking of two zone plates with 100 nm outermost zone period, each with 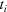 = 1 µm thickness, at 10 keV photon energy. Using the four different stacking schemes shown in Fig. 8 ▸, in (*a*) we show both the radial intensity profile and also the radially integrated energy for 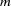 = 2 second diffraction order focusing. The 1:3 line:space approach with the second zone plate being a complementary zone plate has a unique property of having very little energy in sidelobes around the central focus spot. In (*b*) we show the normalized focus intensity for 1:3 line:space stacking with regular and complementary second zone plate as a function of separation distance 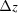, demonstrating a crossover between which approach is preferred at half of 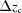 of equation (13). In (*c*) we show the focal spot intensity as the line:space ratio is adjusted in both the upstream and (regular) downstream zone plate; the optimum line:space ratio for a variety of separation distances 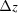 is shown in (*d*).

We consider the second diffraction order (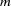 = 2) focusing properties of zone plates with differing line:space ratio in Fig. 9 ▸ where we also consider the option of having either a regular or complementary second zone plate. These simulations were carried out with a thickness of 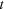 = 1 µm of Au and an incident X-ray energy of 10 keV. In Fig. 9(*a*) ▸, we show the radial focal profiles, and radially integrated intensity, as a function of radius from the optical axis in the case where the outermost zone period is 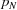 = 100 nm and the separation is 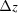 = 50 µm. As can be seen, for second-order focusing a 1:3 line:space ratio is preferred over a 1:1 line:space ratio, as expected (for a thin zone plate, a 1:1 line:space ratio would give zero efficiency for the second-order focus). Having the second zone plate being a regular zone plate gives higher focusing efficiency than using a complementary zone plate.

One very interesting feature of the complementary 1:3 line:space ratio case is that there is very little increase in light in ‘sidelobes’ outside the central focus spot (though the central focus spot is slightly widened). This can be of advantage in methods such as the mapping of trace elements by X-ray fluorescence, since a focal probe without sidelobes will allow for better quantitation of the elemental content within the central focus spot with little or no signal contributed from other nearby positions.

When working in the first diffraction order, downstream stacked zone plates will be of the same type as the first one (that is, regular rather than complementary zone plates) with their parameters modified with separation distance as described in §2. However, when working in the second diffraction order, the wavefield converging from the first zone plate will converge at twice the normal angle, or 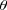 = 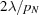, which when multiplied by the separation distance 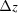 gives a reduction of radius for a given zone number of 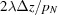 rather than 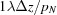. If one sets the *extra* radius reduction of 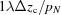 equal to one zone period 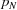, one finds 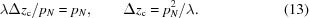
That is, at a distance of 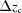 the second zone plate will work with its zones shifted by one period, and at half that distance one will have a transition to requiring that the second zone plate be a complementary one rather than a regular one. For 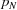 = 100 nm and 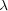 = 0.124 nm corresponding to 10 keV, the ‘matched’ distance is 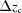 = 81 µm and the transition to a complementary zone plate should occur at half that distance or about 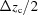 = 40 µm. This effect is shown in Fig. 9(*b*) ▸, where when using stacked 1:3 line:space ratio zone plates in the second diffraction order one finds that a complementary zone plate is preferred at distances smaller than 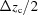, and at 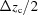 and above there is a change to preferring a regular zone plate.

For two zone plates stacked in close proximity (such as interlaced zone plates) operating in the second diffraction order, a line:space ratio of 1:3 should give near-optimum diffraction efficiency. However, the optimum line:space ratio can be different when two zone plates become more separated. We therefore undertook simulations where the line:space ratio of both the first and second zone plates was adjusted. The results for a fixed separation distance of 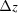 = 50 µm are shown in Fig. 9(*c*) ▸, which indicates that a line:space ratio of about 1:2.7 is preferred to 1:3 for both zone plates in regular stacking. However, given that complementary zone plates can be preferred at certain distances as shown in Fig. 9(*b*) ▸ and as discussed above, in Fig. 9(*d*) ▸ we show the focus intensity for the optimum line:space ratio, and also the value of that optimum line:space ratio, for two stacked zone plates as a function of separation distance 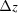.

**Figure 10:**
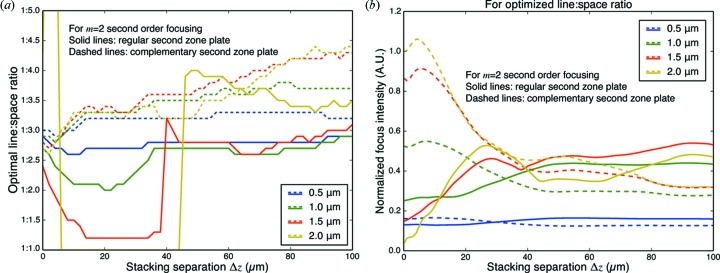
Optimum line:space ratio (*a*) and resulting relative focusing intensity (*b*) for 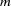 = 2 second-order focusing as a function of both zone thickness 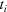 and separation distance 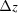. The differences between using a regular or a complementary zone plate for the second optic become larger when thickness 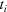 approaches 2 µm which corresponds to a π phase shift.

The calculations shown in Fig. 9 ▸ were for the case of a fixed zone thickness of 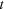 = 1 µm. In Fig. 10 ▸, we show how the optimal line:space ratio depends on both the thickness 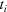 of the two individual zone plates, and the stacking separation distance 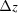. At a zone plate thickness of 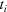 = 0.5 µm, the differences between having the second zone plate being regular or complementary are not so significant, whereas they are quite noticeable with 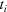 = 2 µm, which corresponds to a π phase shift. This makes it clear that the differences between using a regular or a complementary zone plate for the second zone plate are volume diffraction effects.

## 6. Refocusing zone plates   

**Figure 11:**
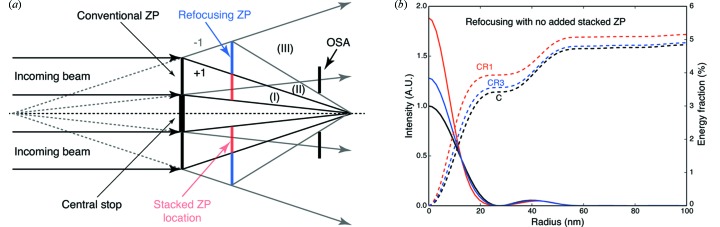
Refocusing zone plates can recapture some of the 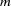 = −1-order focus light from a conventional zone plate. This is shown schematically in (*a*), where one can produce a combined optic with a stacked zone plate in the inner diameter and a refocusing zone plate operating in either 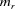 = 1 or first diffraction order, or 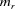 = 3 or third diffraction order. The effect on the focus profile and integrated energy as a function of radius is shown in (*b*), for the cases of a conventional zone plate alone (case C), or with the addition of a refocusing zone plate operating in first diffraction order (case CR1), or with a refocusing zone plate operating in third diffraction order (case CR3). Both refocusing zone plates offer an increase in focal efficiency, with the CR1 case also offering an improvement in spatial resolution. However, in the CR1 case the refocusing zone plate must have finer zone width 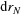 than the conventional zone plate, and usually the conventional zone plate is fabricated out to the limits of what can be achieved in nanolithography; in the CR3 case, the finest zone width 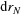 is the same for the conventional (C) and third-order refocusing (R3) zone plates. The parameters for the zone plates used for the calculation of focal intensities (*b*) are given in Table 2 ▸.

In the simulations shown above, only the zeroth or positive focal orders are used from upstream zone plates. However, significant energy goes into negative diffraction orders, which diverge from a virtual focus located upstream. We now consider schemes to recapture a fraction of this energy using a stacked refocusing zone plate. The idea is shown schematically in Fig. 11(*a*) ▸. The refocusing zone plate can be designed to operate in 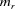 = 1 or first diffraction order, but this requires a finer outermost zone width 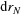 in the refocusing zone plate than is used in the first or second (stacked) zone plate. Therefore another option to consider is the use of 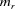 = 3 or third-order diffraction by the refocusing zone plate, so that the minimum zone width 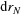 in the refocusing zone plate is larger than the minimum zone width 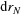 in the conventional and stacked zone plate. For the refocusing zone plate, the usual expression for conventional zone plate zone radii 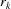 of equation (12) is replaced with 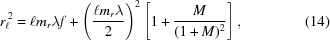
where 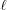 indexes the zones in the refocusing zone plate, and *M* is the magnification of the source to its image.

In order to understand the potential improvements that might be provided by using a refocusing zone plate, we first calculated three cases where we did not include a stacked zone plate: a conventional zone plate with central stop [case C in Fig. 11(*b*) ▸], the conventional zone plate plus a refocusing zone plate operated with 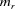 = 1 or first diffraction order [case CR1 in Fig. 11(*b*) ▸], and a conventional zone plate plus a refocusing zone plate operated with 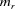 = 3 or third diffraction order [case CR3 in Fig. 11(*b*) ▸]. The parameters of the zone plates considered can be found in Table 2 ▸. This figure demonstrates that the refocusing zone plate can add substantially to the conventional zone plate’s focusing efficiency.

**Figure 12:**
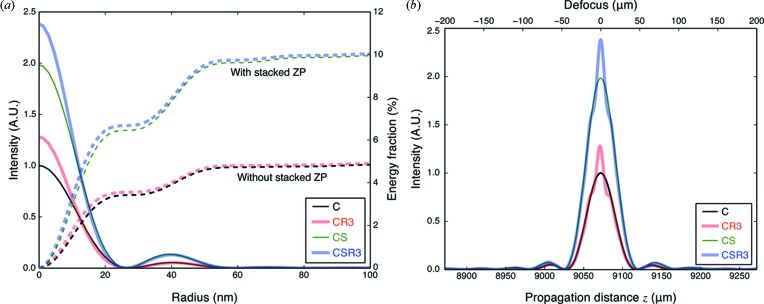
Effect of using both stacked and refocusing zone plates. Four combinations were compared: a conventional zone plate alone (C), or optionally with a stacked zone plate (S), or optionally with a third diffraction order refocusing zone plate (R3). This gives combinations C, CS, CR3 and CSR3 as indicated. On the left (*a*) is shown the focused intensity profile and radial integral of energy, while on the right (*b*) is shown the set of axial intensity profiles. Adding a refocusing zone plate provides some increase in focused intensity, but only over a narrow depth of focus range reduced by a factor of 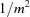 due to the presence of the third diffraction order refocusing zone plate (R3). The parameters for the individual zone plates are shown in Table 3 ▸.

Having seen the effect of a refocusing zone plate alone in Fig. 11(*b*) ▸, we add a stacked zone plate in the position indicated in Fig. 11(*a*) ▸ to give the results shown in Fig. 12 ▸. In this case, a comparison was made between a conventional zone plate (C) with and without a stacked zone plate (S), and with and without a third-order refocusing zone plate (R3), using the parameters shown in Table 3 ▸. As can be seen, the re­focusing zone plate adds to the intensity in the first-order focus, though the stacked zone plate S plays a more important role than the refocusing zone plate R3. We note that the stacked S and refocusing R3 zone plates can be fabricated on the same window, so the refocusing zone plate adds little additional complexity. Also, as the separation distance 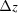 between the conventional zone plate (C) and stacked/refocusing zone plate (SR3) is increased, the area of the stacked zone plate will decrease while the area of the refocusing zone plate will increase and the improvement from using a refocusing zone plate should also increase. Finally, the use of 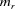 = 3 third-order diffraction in the refocusing zone plate R3 leads to a 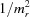 sharpening of the axial intensity profile as shown in Fig. 12(*b*) ▸.

## 7. Conclusion   

Following the introduction of the concept of stacking multiple zone plates at beyond-proximity distances (Vila-Comamala *et al.*, 2013 ▸), we have considered here a variety of design options. Using multislice propagation (Cowley & Moodie, 1957 ▸; Li *et al.*, 2017*a* ▸) to handle the case of wavefield propagation within thicker zone plates, we studied the effects of stacked zone plate parameter design schemes and choice of separation distances. In the supporting information, we also follow prior work on understanding the effects of alignment errors (Vila-Comamala *et al.*, 2013 ▸; Gleber *et al.*, 2014 ▸) by providing a more detailed look at the effects on the focal spot.

Combining multiple zone plates can lead to higher focusing efficiency and focal spots with different characteristics (such as reduced sidelobes), but the design details matter. One can also understand the potential improvements by realizing that the stacking of multiple planar zone plates leads one toward the situation of a volume grating. Separate mounting and aligning of up to five stacked zone plates has already been demonstrated (Gleber *et al.*, 2014 ▸), and this approach with mechanical adjustment has the advantage of allowing one to tune the incident photon energy and then adjust the separation distance between zone plates as required. For single-wavelength operation, one can also use monolithic mounting approaches (Feng *et al.*, 2007 ▸) where multiple zone plates are pre-aligned and fixed in place prior to use. In general, more zone plates are better, but the details matter!

## 8. Related literature   

The following references, not cited in the main body of the paper, have been cited in the supporting information: Pratsch *et al.* (2014 ▸); Simpson & Michette (1983 ▸).

## Supplementary Material

Supporting information. DOI: 10.1107/S1600577518007208/mo5178sup1.pdf

## Figures and Tables

**Table 1:** Parameters for 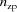 = 4 zone plates stacked at a separation distance of 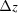 = 1 mm, using the strategies of fixing the diameter *d* in µm, or the number of zones *N*, or the outermost zone width 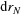 in nm for the *i*th zone plate Under the assumption that a zone plate fabrication process has a limit to achievable aspect ratios 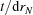, zone plate thicknesses are also adjusted according to 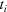 = 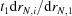 where 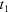 = 500 nm in this case.

	Fixed *d*				Fixed *N*				Fixed 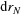			
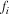 (µm)	*d*	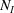	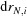	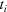	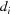	*N*	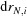	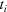	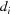	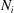	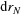	*t*
9074 ( 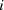 = 1)	45.0	450	25.0	500	45.0	450	25.0	500	45.0	450	25.0	500
8074 ( 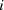 = 2)	45.0	506	22.2	446	42.4	450	23.6	472	40.0	400	25.0	500
7074 ( 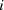 = 3)	45.0	577	19.5	390	39.7	450	22.1	442	35.1	351	25.0	500
6074 ( 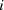 = 4)	45.0	672	16.7	334	36.8	450	20.5	410	30.1	301	25.0	500

**Table 2:** Zone plate parameters used for the calculation shown in Fig. 11(*b*) ▸ In the case of a refocusing zone plate operating in first diffraction order, one can see that a much smaller outermost zone width 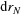 is required than in the conventional zone plate. Because nanolithography processes are often limited in their achievable aspect ratio 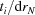, the thickness of R1 was decreased to maintain a limiting aspect ratio of 20.

Zone plate	Label	*d* (µm)	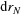 (nm)	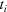 (nm)	*f* (µm)	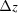 (µm)
Refocusing zone plate (first order)	R1	20–60	8.3	167	4032	3024
Refocusing zone plate (third order)	R3	20–60	25	500	12096	3024
Conventional zone plate	C	15–45	25	500	9074	

**Table 3:** Zone plate parameters assumed for Fig. 12 ▸

	Label	*D* (µm)	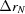 (nm)	*t* (nm)	*f* (µm)
Conventional zone plate	C	15–45	25	500	9074
Stacking zone plate	S	10–30	25	500	6049
Refocusing zone plate	R3	30–60	25	500	12096
