# Supplementary material for: More are better, but the details matter: combinations of multiple Fresnel zone plates for improved resolution and efficiency in X-ray microscopy
Source: J Synchrotron Radiat. 2018 Jun 17;25(Pt 4):1048–59. doi: 10.1107/S1600577518007208 (PMC6038614; doi:10.1107/S1600577518007208)
Supplement: Supplementary file 1 [file s-25-01048-sup1.pdf]

JOURNAL OF SYNCHROTRON RADIATION

Volume 25 (2018)

Supporting information for article:

More are better, but the details matter: combinations of multiple Fresnel zone plates for improved resolution and efficiency in X-ray microscopy

Kenan Li and Chris Jacobsen

Supplementary material to "More are better, but the details matter: combinations of multiple Fresnel zone plates for improved resolution and efficiency in x-ray microscopy"

Kenan Li[a,b1] and Chris Jacobsen [b,c,d*]

[a]*Applied Physics, Northwestern University, Evanston, IL 60208, USA,* [b]*Advanced Photon Source, Argonne National Laboratory, Argonne, IL 60439, USA,* [c]*Department of Physics & Astronomy, Northwestern University, Evanston, IL 60208, USA, and* [d]*Chemistry of Life Processes Institute, Northwestern University, Evanston, IL 60208, USA. E-mail: cjacobsen@anl.gov*

Fresnel zone plates; X-ray microscopy; X-ray optics

In this Supplement, we follow prior work on understanding the effects of alignment errors on stacked zone plates (Vila-Comamala *et al.*, 2013; Gleber *et al.*, 2014) by providing a more detailed look at the effects on the focal spot. This is done by considering the first or upstream zone plate to have a diameter $d = 45$ μm, and an outermost zone width of $dr_N = 25$ nm, thus giving a focal length of $f = 9074$ μm at 10 keV and a depth of focus of $2\delta_z = 25$ μm. Each individual zone plate is assumed to have a thickness of $t = 500$ nm, giving a theoretical first order focusing efficiency of 5.0% at 10 keV for a single zone plate and 32.7% for $n_{\rm zp} = 4$ such zone plates placed in close proximity.

In order to illustrate the effects of larger separation distances between zone plates, Fig. 1 shows $n_{\rm zp} = 4$ of the above zone plates stacked at intervals of $\Delta z = 10$ μm

[1] Present address: SLAC, Menlo Park, CA 94025, USA

(A) so that the net separation between upstream and downstream zone plates is 30 μm, or at intervals of $\Delta z = 100$ μm leading to a net separation of 300 μm (B). The intervals with case (A) are less than the depth of focus of 25 μm, so one obtains a sharp focus with only a 24.8% increase in the FWHM probe width, but with a reduction in efficiency to 15.6% versus the close-proximity case of 32.7%. When the intervals are increased well beyond the depth of focus (B), four distinct axial foci are produced so it is difficult to talk about a common FWHM probe size or a simple focusing efficiency value. However, if one allows the ith zone plate to have its diameter d_i adjusted so as to focus to the same point for all four zone plates, one obtains a fine focus with $\delta_{\mathrm{FWHM}} = 25.4$ nm and a net efficiency of 39.3%.

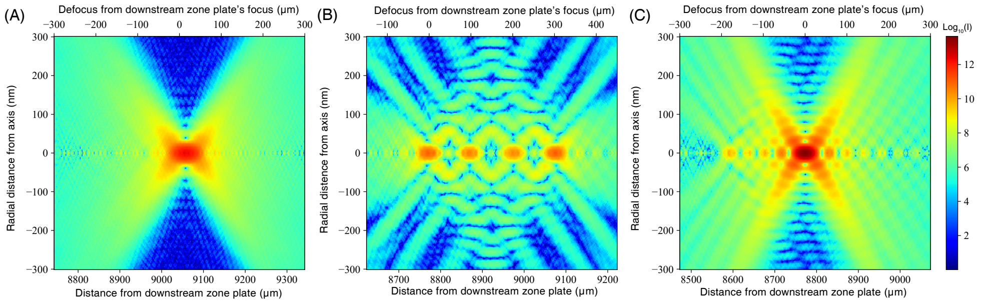

Fig. 1. Simulation of beyond-near-field stacking $n_{\mathrm{zp}} = 4$ gold zone plates. The upstream zone plate is assumed have a diameter of $d = 45$ μm and an outermost zone width of $dr_N = 25$ nm, giving a focal length of 9074 μm at an x-ray energy of 10 keV and a depth of focus of $2\delta_z = 40.3$ μm. If we stack four zone plates (each of $t = 500$ nm thick gold) in close proximity, the theoretical first-diffraction-order focusing efficiency is 32.7%. If we instead stack these zone plates with $\Delta z = 10$ μm separation (A), the diffraction efficiency is reduced to 15.6% and the full-width at half-maximum focal spot size is slightly enlarged from $\delta_{\mathrm{FWHM}} = 26$ nm to 32.2 nm. (B) If the separation is increased to 100 μm which is larger than the depth of focus, four distinct focal points are produced along the beam direction, and the separation between foci is the same as the zone plate separation. (C) If the zone plates are separated by $\Delta z = 100$ μm but the diameter d_i of the ith zone plate is adjusted so that it focuses to the position of the first, upstream zone plate, one obtains a higher efficiency of 39.3% and a tighter focal spot with $\delta_{\mathrm{FWHM}} = 25.4$ nm.

Several studies (Simpson & Michette, 1983; Pratsch *et al.*, 2014) have shown that the focusing properties of single Fresnel zone plates begin to degrade when zones

are mispositioned by about a third to a half of their width (half a zone width or $\sim dr_N/2$ for the outermost zone corresponds to the Rayleigh quarter-wave criterion). One would expect something similar with lateral alignment in zone plate stacking (Shastri *et al.*, 2001; Maser *et al.*, 2002; Snigireva *et al.*, 2007; Kagoshima *et al.*, 2011; Gleber *et al.*, 2014), so that the lateral error Δx_i should be small compared to the outermost zone width dr_N. An additional requirement in zone plate stacking is that the longitudinal error Δz_i should be small compared to the depth of focus DoF, which is most restrictive for the most downstream zone plate due to its finer zone width. These alignment errors are shown in Fig. 2. The role of both errors in zone plate stacking has been examined previously in both simulation and experiment (Gleber *et al.*, 2014).

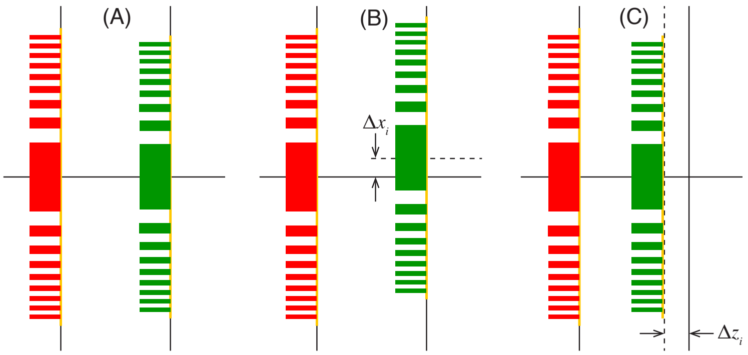

Fig. 2. Zone plate stacking with no error (A), a transverse misalignment Δx_i (B), and a longitudinal misalignment Δz_i (C).

For zone plate transverse misalignment, Fig. 3 shows simulations of the stacking of two zone plates with 50 μm separation and misalignment Δx_i ranging from 0 to $2dr_{N,1}$. We see that a misalignment of $\Delta x_i = 1.0dr_{N,1}$ leads to an elliptical focal spot, while a misalignment of $\Delta x_i = 2.0dr_{N,1}$ leads to split focal spots within the focal plane (the transition to multiple focal spots also leads to fringes in the far-field diffraction pattern (Shastri *et al.*, 2001; Maser *et al.*, 2002; Gleber *et al.*, 2014)). Similar to the

case for zone placement errors in single zone plates, when the transverse misalignment exceeds about $\Delta x_i \sim dr_{N,1}/3$, Fig. 3(D) shows that the peak intensity of the focus begins to decrease and the FHWM spot size begins to increase.

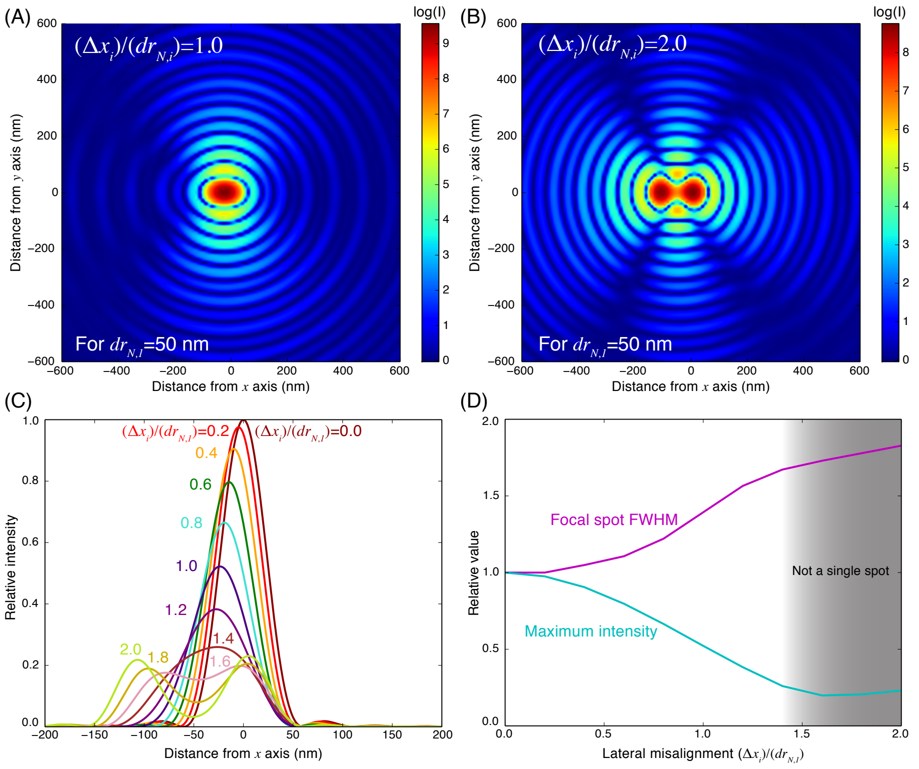

Fig. 3. Transverse misalignment in zone plate stacking. At top is shown the focal spot intensity with a transverse misalignment between two zone plates of $\Delta x_i = 1.0dr_{N,1}$ (A) and $\Delta x_i = 2.0dr_{N,1}$ (B). At bottom left (C) is shown a series of plots of the transverse intensity profile for a range of transverse misalignments Δx_i ranging from $0.0dr_{N,1}$ to $2.0dr_{N,1}$, while at right (D) we show the relative change in both the full-width at half maximum (FWHM) focal spot size, and the maximum intensity. As can be seen, transverse alignment errors Δx_i of about half of the outermost zone width, or $\sim 0.5dr_{N,1}$, lead to degradations in zone plate focusing. These simulations used two zone plates, each with diameter $d = 45$ μm and thickness $t_i = 1$ μm, and an outermost zone width on the first zone plate of $dr_{N,1} = 50$ nm. The separation between the two zone plates was $\Delta z = 50$ μm, and a photon energy of 10 keV was used.

For zone plate longitudinal misplacement, Fig. 4 shows simulations of alignment errors Δz_i ranging from 0 to 1 DoF. We see that a misplacement of $\Delta z_i = 1.0$DoF

leads to an extended focal spot in depth, while a misplacement of $\Delta z_i = 2.0\text{DoF}$ leads to a split focal spot along the longitudinal axis as well as a wider but hollow focal spot at the midpoint. The derivative of a zone plate's focal length f of with respect to diameter d gives

$$\Delta f = (\Delta d)\frac{dr_N}{\lambda} \tag{1}$$

while the depth of focus can be arranged to give $(dr_N/\lambda) = \text{DoF}/(4.88dr_N)$, which when substituted into Eq. 1 gives

$$\frac{\Delta f}{\text{DoF}} = \frac{\Delta d}{4.88dr_N}. \tag{2}$$

If the diameter d is pulled back by half of the outermost zone width dr_N (a Rayleigh quarter-wave deviation) on each side of the optical axis, the net change in diameter is $\Delta d = dr_N$ so $(\Delta f) = \text{DoF}/4.88 \simeq 0.21\text{DoF}$ corresponds to the Rayleigh quarter wave criterion for longitudinal misalignment of the two zone plates. As can be seen in Figs. 4(C) and (D), this gives a slightly over-restrictive tolerance on longitudinal alignment.

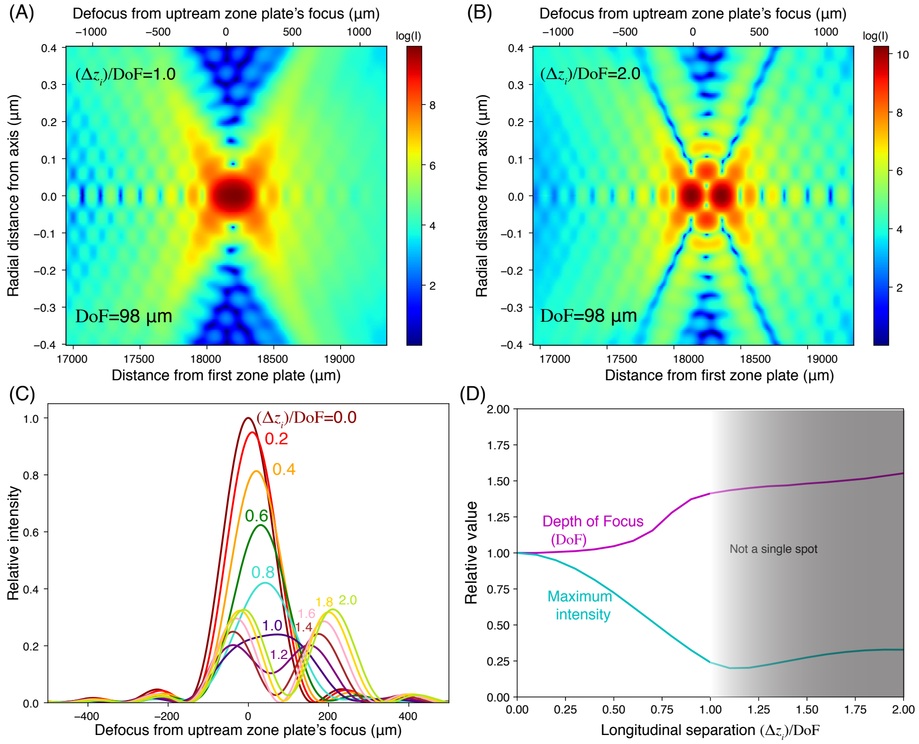

Fig. 4. Longitudinal misalignment in zone plate stacking as a function of depth of focus, which is DoF $= 98.4$ μm in this case. At top is shown the longitudinal-radial intensity distribution for a misalignment of $\Delta z_i = 1.0$DoF (A) and for $\Delta z_i = 2.0$DoF (B); in the latter case, two distinct focal spots appear along the longitudinal axis. At bottom left (C) is shown a series of plots of the focus intensity profile for a range of longitudinal misalignments Δz_i ranging from 0.0DoF to 2.0DoF, while at right (D) we show the relative change in both the full-width at half maximum (FWHM) focal spot size, and the maximum intensity. As can be seen, longitudinal alignment errors Δz_i of about a third of a depth of focus, or ~ 0.3DoF, lead to degradations in zone plate focusing. These simulations used two zone plates, each with diameter $d = 45$ μm and thickness $t_i = 1$ μm, and an outermost zone width on the first zone plate of $dr_{N,1} = 50$ nm. The separation between the two zone plates was $\Delta z = 50$ μm, and a photon energy of 10 keV was used.

With a single thin zone plate or grating with 1:1 line:space ratio, only odd order diffractions and foci are produced. Even-order diffraction (such as second order) comes either from departures from a 1:1 line:space ratio, or volume diffraction effects (dynamic diffraction within gratings); both of these effects are shown in Fig. 5(A). Even-diffraction-order focusing is also increased when using stacked zone plates, as

shown in Fig. 5(B). This might be due to the additional high-spatial-frequency modulation imposed on the wavefield by the second zone plate.

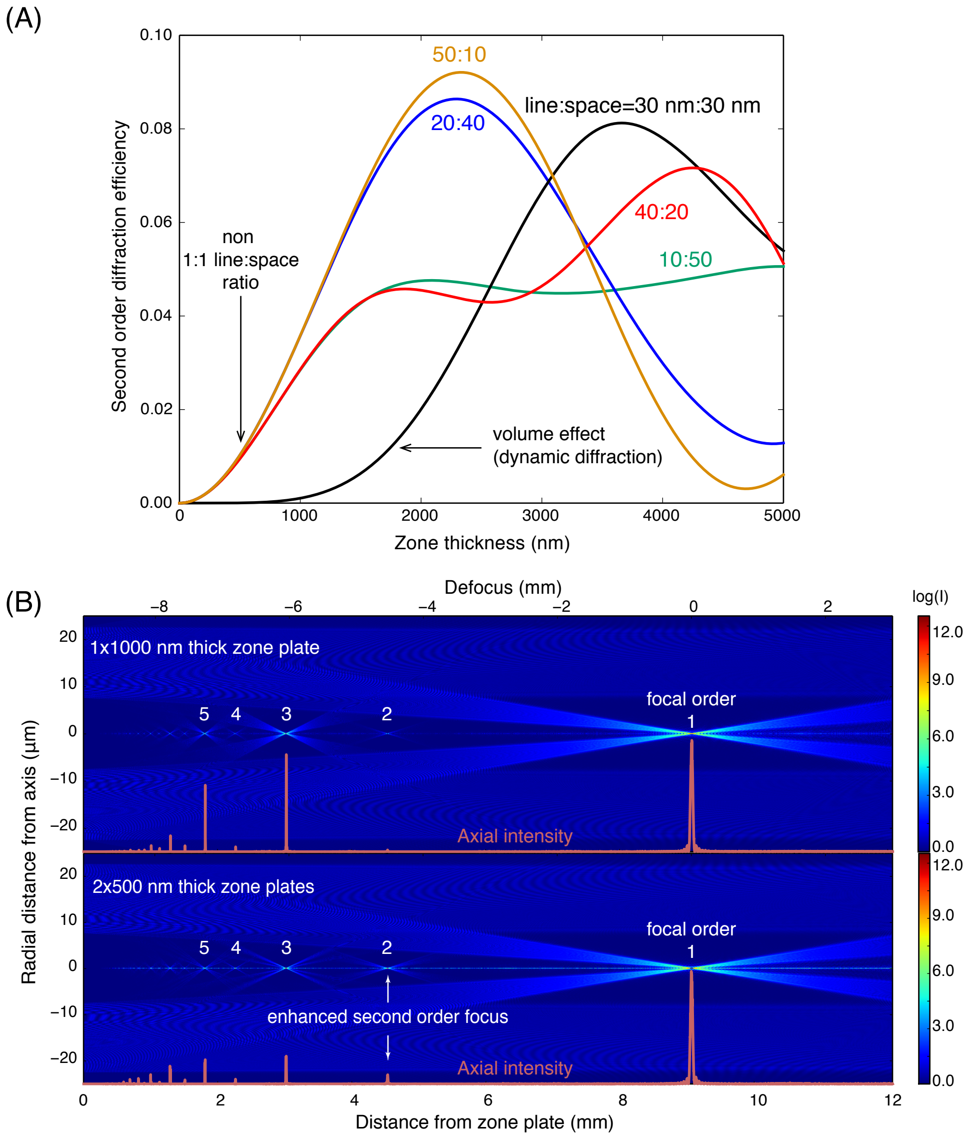

Fig. 5. Thin zone plates with a 1:1 line:space ratio show no second order diffraction. However, light in a second order focus can appear (A) when the line:space ratio is changed from 1:1, but also when volume diffraction effects begin to play a role (as calculated using the multislice method (Li *et al.*, 2017)). Light in the second order focus can also appear when one uses stacked zone plates. Shown here (B) is a comparison between a single $t = 1000$ nm thick zone plate, and two 500 nm thick zone plates separated by 50 μm. As before, these simulations are for $d = 45$ μm diameter zone plates with $dr_N = 25$ nm outermost zone width and (for B) a line:space ratio of 1:1. An x-ray energy of 10 keV was used.

References

Gleber, S. C., Wojcik, M., Liu, J., Roehrig, C., Cummings, M., Vila-Comamala, J., Li, K., Lai, B., Shu, D. & Vogt, S. (2014). *Optics Express*, **22**(23), 28142–28153.

Kagoshima, Y., Takano, H., Koyama, T., Tsusaka, Y. & Saikubo, A. (2011). *Japanese Journal of Applied Physics*, **50**(2).

Li, K., Wojcik, M. & Jacobsen, C. (2017). *Optics Express*, **25**(3), 1831–1846.

Maser, J., Lai, B. P., Yun, W., Shastri, S. D., Cai, Z., Rodrigues, W., Xu, S. & Trackhtenberg, E. (2002). *Proceedings SPIE*, **4793**, 74–81.

Pratsch, C., Rehbein, S., Werner, S. & Schneider, G. (2014). *Optics Express*, **22**(25), 30482–30491.

Shastri, S. D., Maser, J. M., Lai, B. & Tys, J. (2001). *Optics Communications*, **197**(1-3), 9–14.

Simpson, M. J. & Michette, A. G. (1983). *Optica Acta*, **30**(10), 1455–1462.

Snigireva, I., Snigirev, A., Kohn, V., Yunkin, V., Grigoriev, M., Kuznetsov, S., Vaughan, G. & Di Michiel, M. (2007). *Physica Status Solidi A*, **204**(8), 2817–2823.

Vila-Comamala, J., Wojcik, M., Diaz, A., Guizar-Sicairos, M., Kewish, C. M., Wang, S. & David, C. (2013). *Journal of Synchrotron Radiation*, **20**(3), 397–404.
